# *VEGFA* rs3025039 is associated with phenotype severity of myelofibrosis‐type megakaryocyte dysplasia

**DOI:** 10.1002/jha2.708

**Published:** 2023-05-07

**Authors:** Giovanni Barosi, Paolo Catarsi, Rita Campanelli, Margherita Massa, Adriana Carolei, Carlotta Abbà, Annalisa de Silvestri, Robert Peter Gale, Vittorio Rosti

**Affiliations:** ^1^ Center for the Study of Myelofibrosis General Medicine 2 Istituto di Ricovero e Cura a Carattere Scientifico Policlinico S. Matteo Foundation Pavia Italy; ^2^ Center for Systemic Amyloidoses and High Complexity Diseases General Medicine 2 Istituto di Ricovero e Cura a Carattere Scientifico Policlinico San Matteo Foundation Pavia Italy; ^3^ Biometry and Clinical Epidemiology Scientific Direction Istituto di Ricovero e Cura a Carattere Scientifico Policlinico San Matteo Foundation Pavia Italy; ^4^ Department of Immunology and Inflammation Centre for Haematology Research Imperial College London London UK

1

BCR::ABL‐negative myeloproliferative neoplasms (MPNs) include the World Health Organization (WHO) defined classical MPNs, that is essential thrombocythaemia, polycythaemia vera (PV) and primary myelofibrosis (PMF). Recently, two new cognate phenotypes, that is, clonal megakaryocyte dysplasia with normal blood values (CMD‐NBV), and clonal megakaryocyte dysplasia with isolated thrombocytosis (CMD‐IT) have been proposed [1, 2]. These entities, along with prefibrotic myelofibrosis (pre‐MF) and overt‐MF were unified by bone marrow (BM) myelofibrosis‐type megakaryocyte dysplasia (MTMD) [3].

Myeloproliferation in MPNs is driven by *gain‐of‐function* mutations in *JAK2*, *CALR* or *MPL* genes. Persons with these mutations may have additional mutations common to other myeloid cancers including *ASXL1, EZH2, DMNT3A, IDH1* and *IDH2*. However, these divergent genotypes do not completely account for different MPN phenotypes and new explanations are sought.

Single nucleotide variations (SNVs) are a possible explanation for this phenotypic heterogeneity. Three common SNVs of *VEGFA*, that is, rs2010963, rs3025020 and rs3025039, influence predisposition, phenotype, and risk of thrombosis in subjects with PMF [4, 5, 6]. We interrogated a possible association between the *VEGFA* SNVs and variant phenotypes in the MTMD category. We analyzed stored DNA from blood granulocytes of 857 consecutive subjects seen at the Center for the Study of Myelofibrosis of the IRCCS Policlinico S. Matteo Foundation in Pavia. The cohort included 23 subjects with CMD‐NBV,119 with CMD‐IT, 349 with pre‐MF and 366 with overt‐MF (Table S1).

Subjects were classified as pre‐MF or overt‐MF based on operative WHO diagnostic criteria at the time of their first visit and re‐classified according to 2022 revised criteria. Subjects were classified as CMD‐NBV or CMD‐IT based on adjudicated criteria [1, 2]. All subjects gave written informed consent approved by the IRCCS Policlinico S. Matteo Foundation Institutional Ethics Committee for their data to be collected and stored in the database of the Center for the Study of Myelofibrosis. The Ethics Committee of the Hospital also approved a written informed consent for patients to donate samples for molecular research (Reference 20110004143 of 26 September 2011). Controls were healthy Italians participating in a BM donor transplant registry whose samples were made anonymous for the purpose of the study (*N* = 201).

Details on the genotyping process are described [4, 5, 6]. In all cohorts of diseased subjects and controls, *VEGFA* SNVs did not deviate from Hardy‐Weinberg equilibrium, revealing the reliability of the study samples. First, we investigated the minor allele (MAF) and the genotype frequencies of the three *VEGFA* SNVs in the MTMD population (Table S2). *VEGFA* rs2010963 and rs3025020 MAF were similar in subjects and controls. In contrast, the *VEGFA* rs3025039 MAF was lower in subjects compared with controls (16% vs. 20%; odds ratio [OR] = 0.77; 95% confidence interval [CI], 0.59, 1.02; *p* = 0.08). In the rs2010963 and rs3025020 carrying at least one minor allele, GC‐CC or CT‐TT (dominant model), resulting in an OR = 1 (0.73, 1.37); *p* = 0.99, and OR = 1.21(0.89, 1.64), *p* = 0.22. In contrast, OR resulted in 0.75 (0.54, 1.08); p= 0.08 for rs3025039. Although no statistical differences were obtained, these results suggest a potential association between the *VEGFA* rs3025039 genotype and the risk of MTMD, implying the minor T allele of the SNV is protective against developing MTMD variants.

Next, we studied MAF and genotype frequencies of *VEGFA* SNVs in subjects with MTMD stratified for the four aforementioned phenotypes ordering them by severity from CMD‐NBV (less severe) to overt‐MF (more severe) passing through CMD‐IT and pre‐MF. *VEGFA* rs2010963 and rs302520 had no MAF or genotype frequencies significantly associated with any MTMD phenotypes (data not shown). In contrast, we found a negative association between *VEGFA* rs3025039 MAF and CT‐TT genotypes (dominant model) with the four MTMD phenotypes (Table 1). Compared with controls, the OR of MAF was 0.39 (0.13, 1.12; *p* = 0.08) in CMD‐NBV, OR = 0.57 (0.36, 0.89; *p* = 0.016) in CMD‐IT, OR = 0.72 (0.52, 0.99; *p* = 0.49) in pre‐MF, and OR = 0.94 (0.69, 1.28) *p* = 0.70 in overt‐MF. *VEGFA* rs3025039 CT‐TT genotypes were identified in four of 23 (17%) subjects with CMD‐NBV, 28 of 119 (24%) with CMD‐IT, 97 of 349 (28%) with pre‐MF, and 121 of 366 (33%) with overt‐MF, compared with 71 of 201 controls (35%). Differences in the CT‐TT genotypes between subjects and controls were most pronounced in CMD‐NBV and CMD‐IT (*p* = 0.028 and *p* = 0.09). MAF and CT‐TT genotype frequencies increased from the less to more severe MTMD phenotypes (*p*‐value for trend = 0.031, χ2 test, 8.87 and *p*‐value for trend = 0.09, χ2 test, 6.37, respectively). These data support the hypothesis the T allele of the *VEGFA* rs3025039 SNV protects against the occurrence of lesser severe MTMD phenotype variants.

**TABLE 1:** Allele frequency and frequency of the genotypes of the rs3025039 *VEGFA* single nucleotide variation (SNV) in the myelofibrosis‐type megakaryocyte dysplasia (MTMD) stratified for the phenotypic variants and comparison with normal control subjects.

**Model**	**Genotype**	**Case *N* (%)**	**Control N (%)**	**OR (95% CI)**	**p‐Value**
*Clonal megakaryocyte dysplasia with normal blood values (CMD‐NBV)*					
Allele	C	42 (91.3)	323 (80.3)	1	
	T	4 (8.7)	79 (19.7)	0.39 (0.13–1.12)	0.079
Dominant	CC	19 (82.6)	130 (64.7)	1	
	CT‐TT	4 (17.4)	71 (35.3)	0.38 (0.13–1.18)	0.094
*Clonal megakaryocyte dysplasia with isolated thrombocytosis (CMD‐IT)*					
Allele	C	209 (87.8)	323 (80.3)	1	
	T	29 (12.2)	79 (19.7)	0.57 (0.36–0.89)	0.0157
Dominant	CC	91 (76.5)	130 (64.7)	1	
	CT‐TT	28 (23.5)	71 (35.3)	0.56 (0.34–0.94)	0.028
*Prefibrotic myelofibrosis (Pre‐MF)*					
Allele	C	593 (85)	323 (80.3)	1	
	T	105 (15)	79 (19.7)	0.72 (0.52–0.99)	0.49
Dominant	CC	252 (72.2)	130 (64.7)	1	
	CT‐TT	97 (27.8)	71 (35.3)	0.70 (0.48–1.02)	0.065
*Overt myelofibrosis (Overt‐MF)*					
Allele	C	595 (81.3)	323 (80.3)	1	
	T	137 (18.7)	79 (19.7)	0.94 (0.69–1.28)	0.70
Dominant	CC	245 (66.9)	130 (64.7)	1	
	CT‐TT	121 (33.1)	71 (35.3)	0.90 (0.63–1.30)	0.58

To take account for co‐variates implicated in the severity of the MTMD phenotypic variants, including age, sex, and *CALR* mutations, we applied a multivariable proportional odds model to test for the association between MTMD phenotypes and *VEGFA* rs3025039 SNV frequency. We found that male sex (adjusted OR = 1.53 [1.17, 2.01]; *p* = 0.002), older age [adjusted OR = 1.05 per 1‐year increase (1.04, 1.06); *p* < 0.001], *CALR* mutation (adjusted OR = 1.88 [1.02, 3.49]; *p* = 0.043) were all significantly associated with MTMD severity, consistent with previous findings [1, 2]. However, even after adjusting for these factors, the presence of CT‐TT genotypes of rs3025039 was associated with an increased risk of more severe MTMD phenotypes (OR = 1.39 [1.08, 1.79]; *p* = 0.011). The proportional odds assumption was tested with the likelihood ratio test and the Brant method (*p* = 0.45 and *p* = 0.35 respectively).[7] These data reinforce the concept of MPNs as a spectrum of disorders promoted by specific constitutive features.

Interest in associations between *VEGFA* SNVs and MTMD variant severities is based on the possible myeloproliferation‐favouring properties of VEGFA encoded growth factor via a postulated impact on immune dysfunction and dysregulated angiogenesis.[8] The *VEGFA* rs3025039 variant maps to +936 nucleotide position in the 3′‐UTR resulting in loss of a potential binding site for transcription factor AP‐4, required for *VEGFA* expression.

*VEGFA* rs3025039 is associated with diverse diseases, including cancers. However, the T allele was found to protect against cancer in some cases [9, 10], and in other cases to be associated with susceptibility [11, 12, 13]. The T allele has been observed to give rise to lower circulating VEGFA levels compared with the C allele [14]. However, studies on a South Italy population have demonstrated that the rs3025039 C/T associated VEGFA levels are variable [15].

In summary, our data provide a foundation to study the mechanism of VEGFA in the occurrence and development of the different forms of MTMD category.

## AUTHOR CONTRIBUTIONS

Giovanni Barosi designed the project; Vittorio Rosti, Margherita Massa, Rita Campanelli, Adriana Carolei, and Carlotta Abbà led the database sample collection and clinical characterization efforts; Paolo Catarsi did genotyping for the dataset; Giovanni Barosi and Annalisa de Silvestri did the statistical analysis of the data. Giovanni Barosi and Robert Peter Gale prepared the typescript. All authors reviewed and approved the typescript. All authors have read and agreed to the published version of the manuscript.

## CONFLICT OF INTEREST STATEMENT

Robert Peter Gale is a consultant to BeiGene Ltd., Fusion Pharma LLC, LaJolla NanoMedical Inc., Mingsight Parmaceuticals Inc., Kite Pharma and CStone Pharmaceuticals; Advisor to Antegene Biotech LLC, Medical Director and FFF Enterprises Inc.; Partner in AZACA Inc.; Board of Directors, RakFond Foundation for Cancer Research Support; Scientific Advisory Board, StemRad Ltd.

## FUNDING INFORMATION

Supported by AIRC 5 × 1000 call “Metastatic disease: the key unmet need in oncology” to MYNERVA project, #21267 (MYeloid Research Venture AIRC); by Ricerca Corrente IRCCS Policlinico San Matteo Foundation, Pavia, Italy, project number 874, code number 08054517, received by Vittorio Rosti  (www.sanmatteo.org).

## ETHICS STATEMENT

The Ethics Committee of the Hospital also approved a written informed consent for patients to donate samples for molecular research (Reference 20110004143 of 26 September 2011).

## Supporting information



Supporting Information

## Data Availability

The data presented in this study are available on request from the corresponding author.
