# Supplementary material for: VEGFA rs3025039 is associated with phenotype severity of myelofibrosis‐type megakaryocyte dysplasia
Source: EJHaem. 2023 May 7;4(3):756–9. doi: 10.1002/jha2.708 (PMC10435675; doi:10.1002/jha2.708)
Supplement: Supplementary file 1 — Supporting Information [file JHA2-4-756-s001.docx]

**Supplemental Table 1.** Baseline co-variates of subjects with myelofibrosis-type megakaryocyte dysplasia (MTMD) stratified according to the phenotype as clonal megakaryocyte dysplasia with normal blood values (CMD-NBV), clonal megakaryocyte dysplasia with isolated thrombocytosis (CMD-IT), prefibrotic myelofibrosis (pre-MF) and overt myelofibrosis (overt-MF)

|  | **CMD-NBV** | **CMD-IT** | | | **pre-MF** | | | **overt-MF** |
| --- | --- | --- | --- | --- | --- | --- | --- | --- |
| **Demographic co-variates** | | | | | | | | |
|  | N = 23 | | N = 119 | | | N = 349 | | N = 366 |
| Age, yrs, median (IQR) | 44 (36-52) | | 42 (34-53) | | | 48 (36-58) | | 58 (49-69) |
| Sex female, *N* (%) | 12 (52) | | 67 (56) | | | 160 (46) | | 109 (30) |
| **Laboratory co-variates** | | | | | | | | |
| Haemoglobin, g/L, median (IQR) | N = 23  135 (127-151) | | N = 119  140 (132-152) | | | N = 346  140 (125-157) | | N = 366  113 (91.5-131) |
| WBC x 10E+9/L, median (IQR) | N = 23  6.7 (5.8-8.8) | | N = 119  8.1 (6.8-9) | | | N = 343  9.4 (7.2-12.4) | | N = 363  8.0 (5.2-12.1) |
| Platelets concentration x10E+9/L, median (IQR) | N = 23  277 (213-385) | | N =119  707 (602-846) | | | N = 343  532 (343-749) | | N = 364  287 (134-522) |
| Spleen index, cm x E+2, median (IQR) | N = 23  90 (90-90) | | N = 119  90 (90-90) | | | N = 340  120 (90-150) | | N = 364  140 (120-200) |
| **IPSS score** | | | | | | | | |
|  | N = 22 | | | N = 111 | | | N = 308 | N = 316 |
| Low, *N* (%) | 21 (95.5) | | | 103 (92.8) | | | 231 (75) | 112 (35.4) |
| INT-1, *N* (%) | *1 (4.5)* | | | 8 (7.2) | | | 46 (14.9) | 71 (22.5) |
| INT-2*, N* (%) | *0 (0)* | | | 0 (0) | | | 24 (7.8) | 74 (23.4) |
| High, *N* (%) | *0 (0)* | | | 0 (0) | | | 7 (2.3) | 59 (18.7) |
| **Biological co-variates** | | | | | | | | |
| Plasma LDH, x ULN, median (IQR) | N = 15  0.83 (0.66-1.00) | | | N = 70  0.82 (0.69-1) | | | N = 182  1.07 (0.85-1.48) | N = 223  1.81 (1.32-2.43) |
| Serum cholesterol, mg/dL, median (IQR) | N = 16  179 (139-208) | | | N = 70  181.5 (160-222) | | | N = 163  158 (129-178) | N = 220  143 (119-175) |
| Blood CD34-positive cells x 10E+6/L, median (IQR) | N = 23  2.06 (1.6 – 3.31) | | | N = 70  4.6 (2.8-7.8) | | | N = 174  6.9 (3.7-23.8) | N = 239  39 (12.4-111) |
| **Bone Marrow Fibrosis Grade** | | | | | | | | |
|  | N = 23 | N = 119 | | | | N = 349 | | N = 366 |
| Grade 0 , *N* (%) | 19 (82) | 77 (65) | | | | 149 (43) | | 0 (0) |
| Grade 1, N (%) | 4 (18) | 42 (35) | | | | 200 (57) | | 0 (0) |
| Grade 2, N (%) | 0 (0) | 0 (0) | | | | 0 (0) | | 263 (72) |
| Grade 3, n (%) | 0 (0) | 0 (0) | | | | 0 (0) | | 103 (28) |
| **Molecular Co-Variates** | | | | | | | | |
|  | N =23 | N = 119 | | | | N = 349 | | N = 366 |
| *JAK2*^V617F^-positive, N (%) | 17 (74) | 66 (55) | | | | 268 (77) | | 232 (63) |
| *CALR* mutation, N (%) | 0 (0) | 44 (37) | | | | 48 (14) | | 73 (20) |
| *MPL* mutation, N (%) | 0 (0) | 5 (4) | | | | 12 (3) | | 26 (7) |
| *Tripe negative*, N (%) | 6 (26) | 4 (4) | | | | 21 (6) | | 35 (10) |

Spleen index was calculated by multiplying the length of the longitudinal axis by the transverse axis of the spleen (manually)

Abbreviations: IQR = Interquartile range; ULN = upper limit of normal; LDH = lactic dehydrogenase.

**Supplemental Table 2.** Allele frequency and frequency of the genotypes of the three *VEGFA* SNVs in the myelofibrosis-type megakaryocyte dysplasia (MTMD) overall population and comparison with normal control subjects.

| SNP ID | Model | Genotype | Case N (%) | Control N (%) | OR (95% CI) | *P*-value |
| --- | --- | --- | --- | --- | --- | --- |
| rs2010963 | Allele | G | 1051 (60.9) | 246 (60.9) | 1 |  |
|  |  | C | 675 (39.1) | 158 (39.1) | 1 (0.80-1.20) | 0.99 |
|  | Codominant | GG | 325 (37.7) | 76 (37.6) | 1 |  |
|  |  | CC | 137 (15.8) | 32 (15.8) | 0.99 (0.63-1.5) | 0.99 |
|  |  | GC | 401 (46.5) | 94 (46.6) | 1 (0.71-1-42) | 0.99 |
|  | Dominant | GG | 325 (37.7) | 76 (37.6) | 1 |  |
|  |  | GC-CC | 538 (62.3) | 126 (62.4) | 1 (0.73-1.37) | 0.99 |
|  | Recessive | GG-GC | 726 (84.1) | 170 (84.2) | 1 |  |
|  |  | CC | 137 (15.9) | 32 (15.8) | 0.99 (0.65-1.52) | 0.99 |
| rs3025020 | Allele | C | 1187 (69.1) | 288 (71.6) | 1 |  |
|  |  | T | 531 (30.9) | 114 (28.4) | 1.13 (0.88-1.43) | 0.31 |
|  | Codominant | CC | 412 (48.0) | 106 (52.7) | 1 |  |
|  |  | TT | 84 (9.8) | 19 (9.5) | 1.13 (0.66-1.96) | 0.64 |
|  |  | CT | 363 (42.2) | 76 (37.8) | 1.23 (0.88-1.72) | 0.216 |
|  | Dominant | CC | 412 (48.0) | 106 (52.7) | 1 |  |
|  |  | CT-TT | 447 (52.0) | 95 (47.3) | 1.21 (0.89-1.64) | 0.22 |
|  | Recessive | CC-CT | 775 (90.2) | 182 (90.5) | 1 |  |
|  |  | TT | 84 (9.8) | 19 (9.5) | 1.03 (0.61-1.75) | 0.88 |
| rs3025039 | Allele | C | 1452 (84.0) | 323 (80.3) | 1 |  |
|  |  | T | 276 (16.0) | 79 (19.7) | 0.77 (0.59-1.02) | 0.075 |
|  | Codominant | CC | 613 (70.9) | 130 (64.7) | 1 |  |
|  |  | TT | 25 (2.9) | 8 (4.0) | 0.66 (0.29-1.50) | 0.324 |
|  |  | CT | 226 (26.2) | 63 (31.3) | 0.76 (0.54-1.06) | 0.112 |
|  | Dominant | CC | 613 (70.9) | 130 (64.7) | 1 |  |
|  |  | CT-TT | 251 (29.1) | 71 (35.3) | 0.75 (0.54-1.08) | 0.082 |
|  | Recessive | CC-CT | 839 (97.1) | 193 (96.0) | 1 |  |
|  |  | TT | 25 (2.9) | 8 (4.0) | 0.77 (0.32-.62) | 0.425 |
